# Data on ectoparasites infestation on small mammals from different habitats in east-coast Peninsular Malaysia

**DOI:** 10.1016/j.dib.2020.105621

**Published:** 2020-04-28

**Authors:** Nur Izzah Izzati Ahmad, Noor Aisyah A Rahim, Azuan Roslan, Madinah Adrus, Mariana Ahamad, Marina Hassan, Muhamad Safiih Lola, Mohd Noor Afiq Ramlee, Muhamad Aidil Zahidin, Mohd Tajuddin Abdullah

**Affiliations:** a Institute of Tropical Biodiversity and Sustainable Development, Universiti Malaysia Terengganu, 21030 Kuala Nerus, Terengganu, Malaysia; b Biodiversity and Forestry Management Division, Ministry of Energy and Natural Resources, Wisma Sumber Asli, 25, Persiaran Perdana, Presint 4, 62574 Putrajaya, Malaysia; c Animal Resource Science and Management Programme, Faculty of Resources Science and Technology, Universiti Malaysia Sarawak, 94300 Kota Samarahan, Sarawak, Malaysia; d Unit of Acarology, Infectious Diseases Research Centre, Institute for Medical Research, National Institute of Health, 40170 Setia Alam, Selangor, Malaysia; e Faculty of Science and Marine Environment, Universiti Malaysia Terengganu, 21030, Kuala Nerus, Terengganu, Malaysia; f Faculty of Ocean Engineering Technology and Informatics, Universiti Malaysia Terengganu, 21030 Kuala Nerus, Terengganu, Malaysia; g Department of Haematology, School of Medical Sciences, Universiti Sains Malaysia Health Campus, 16150 Kubang Kerian, Kelantan, Malaysia

**Keywords:** Bat, Rodent, Mite, Chigger, Lice, Bug, Tasik Kenyir, Setiu Wetland, Off coast-islands, Terengganu, South China Sea

## Abstract

This data article presents on the ectoparasites infestation on small mammals in Peninsular Malaysia. The dataset on ectoparasites infestation is important because it raises a major medical concern regarding the spread of potentially zoonotic disease from wildlife to human. Tick and chigger are the primary ectoparasites as reservoirs of vector-borne diseases found on small mammals in Malaysia. These small mammals that are infested with ectoparasites occupy various types of habitats, including human settlements, could be of community health risks as the carriers of potentially zoonotic diseases. Field samplings were conducted from February 2015 to February 2016 in three different ecological habitats of mixed dipterocarp forest, coastal forest and insular forest, in Terengganu, Malaysia. A total of 35 and 22 species of bats and rodents respectively were captured and examined for ectoparasites. Twenty-three species of bats and 16 species of small mammal were recorded as hosts for at least one species of ectoparasites. These findings show that the highest ectoparasite infestation occurred on bat community.

Specification Table

**Table:** 

Subject area	Biology and ecology
More specific subject area	Ectoparasites
Type of data	Table
	Figure
How data were acquired	Field sampling
Data format	Raw and semi-analysed.
Parameters for data collection	Habitat differences, bats and non-volant small mammals.
Description of data collection	Field samplings and capture small mammals were conducted from February 2015 to February 2016 by using standard mist nets, four-bank harp traps and baited cage traps. Small mammals were restrained in cloth bags, identified, examined for ectoparasite and released at trapping sites. Ectoparasites were removed from the host animal fur by using a fine tooth-comb. Ectoparasites dropped from combing were collected by using the wet sharpen applicator stick and preserved in vials containing 70 % ethanol and labelled separately. The ectoparasites were mounted on slides and identified according to their taxonomic groups.
Data source location	1. Hulu Terengganu, Tasik Kenyir Tanjung Mentong (4° 54′ 08.9” 102° 43′ 28.0”), Sungai Buweh Waterfall (5^°^08’48.0” 102^°^48’02.5”), Belukar Bukit (4°54′24.4” 102°59′08.”), Sekayu Waterfall (4°57’41.4” 102°57’08.3”), Hulu Telemong Forest Reserve (5° 13’ 48” 102° 50’ 8.88”), Kenyir Research Station (05°08’59.5” 102°45’48.9”), Belukar Bukit Waterfall (4° 53’ 25.362” 102° 59’ 33.506”), Taman Pertanian Sekayu (4° 58’ 177” 102° 57’ 467”), Kampung Kemat (5° 00’52.2” 102° 57’ 10.409”), Saok Waterfall (5° 05′ 2.49" 102° 46′47.7") 2. Setiu Wetlands Kampung Limau Nipis (5° 40′ 38.779" 102° 42′ 35.092"), Kampung Fikri (5° 39′ 19.4" 102° 44′ 8.2"),Kampung Gong Batu (5° 39′ 23.1"102° 43′ 18.6"), FRIM Setiu (5°33′58.9" 102°51′17.9"), Peladang Agro (5° 35′ 37.918" 102° 40′ 42.186"), Laguna Agro (5° 41’ 42.589” 102 ° 41’ 59.853”) 3. Off coast islands, South China Sea Pulau Bidong (5°37’15.7” 103°03’28.2”), Pulau Perhentian (5° 54’ 9.767” 102° 45’ 21.283”)
Data accessibility	With the article
Related research article	Ahmad, N.I.I., The species composition and the specialisation degree of ectoparasite found infesting small mammals at Setiu, Hulu Terengganu and off coast islands of Terengganu, (MSc thesis), Universiti Malaysia Terengganu, Kuala Nerus, 2020. [1]

## Value of the data

• The data is useful in providing the information regarding the ectoparasite species composition and the relationship among the ectoparasites with their small mammal hosts.
• The data is valuable for further research to determine whether there are any spatial and temporal changes in ectoparasites composition due to ecological disturbances or climate change.
• The data on the occurrence of the ectoparasite-vector are useful in epidemiology study to predict the potential of presence of the zoonotic disease in the studied area.
• The data also provide information on the interaction of ectoparasites and small mammal hosts in term of the degree of the specificity and distribution of each ectoparasites species.
• The data is vital for the community health and wildlife authorities to monitor the host populations near rural villages and forest edges that maybe the cause of zoonotic diseases spillover.

## 1 Data Description

Systematic field samplings were conducted in three different habitats that are mixed dipterocarp forest, coastal forest and insular forest of Terengganu from February 2015 to February 2016. Table 1 shows the description of 18 selected sampling sites in Terengganu. This data recorded 57 species of small mammals comprising 15 families and six orders (Supplementary Table 1). Out of 1,015 individuals small mammals examined, 289 were infested with eight groups of ectoparasites, which are bat flies, fleas, ticks, mesostigmatids, chiggers, fur mites, bugs and lice (Table 2; Supplementary Tables 2-4). The infestation of ectoparasites was then divided and arranged according to the species of small mammal hosts (Supplementary Tables 5-7).

**Table 1:** List of 18 sampling sites and habitat types in Terengganu, Malaysia.

	Location	Code	Coordinate	Forest type

Hulu Terengganu, Tasik Kenyir				
1	Tanjung Mentong	TMK	4° 54′ 08.9" 102° 43′ 28.0"	Mixed dipterocarp forest
2	Sungai Buweh Waterfall	SBW	5^°^08’48.0” 102^°^48’02.5”	Mixed dipterocarp forest
3	Belukar Bukit	BBK	4°54′24.4" 102°59′08.8"	Mixed dipterocarp forest
4	Sekayu Waterfall	SWK	4°57’41.4” 102°57’08.3”	Mixed dipterocarp forest
5	Hulu Telemong Forest Reserve	KBK	5° 13’ 48” 102° 50’ 8.88”	Mixed dipterocarp forest
6	Kenyir Research Station	KRS	05°08’59.5” 102°45’48.9	Mixed dipterocarp forest
7	Belukar Bukit Waterfall	BBW	4° 53’ 25.362” 102° 59’ 33.506”	Mixed dipterocarp forest
8	Taman Pertanian Sekayu	TPS	4° 58’ 177” 102° 57’ 467”	Mixed dipterocarp forest
9	Kampung Kemat	PLR	5° 00’52.2” 102° 57’ 10.409”	Mixed dipterocarp forest
10	Saok Waterfall	SWF	5° 05′ 2.49" 102° 46′47.7"	Mixed dipterocarp forest
Setiu Wetlands				
11	Kampung Limau Nipis	KLNS	5° 40′ 38.779" 102° 42′ 35.092"	Coastal forest
12	Kampung Fikri	KFS	5° 39′ 19.4" 102° 44′ 8.2"	Coastal forest
13	Kampung Gong Batu	TBS	5° 39′ 23.1"102° 43′ 18.6"	Coastal forest
14	FRIM Setiu	FRIM	5°33′58.9" 102°51′17.9"	Coastal forest
15	Peladang Agro	PAS	5° 35′ 37.918" 102° 40′ 42.186"	Mixed dipterocarp forest
16	Laguna Agro	LAS	5° 41’ 42.589” 102 ° 41’ 59.853”	Coastal forest
Off coast islands, South China Sea				
17	Pulau Bidong	PBS	5°37’15.7” 103°03’28.2”	Island forest
18	Pulau Perhentian	PPB	5° 54’ 9.767” 102° 45’ 21.283”	Island forest

**Table 2:** Summary of habitats, infested small mammals and ectoparasite prevalence recorded in Terengganu, Malaysia

Habitat	Small mammals		Individual examined	Individual infested	Ectoparasite prevalence							
					Bat flies	Fleas	Tick	Mesostigmatid	Chigger	Fur mite	Bug	Lice

Mixed dipterocarp forest	Volant	Total no. of individuals	396	103	75	8	5	13	16	9	1	
		Total no. of species	33	20	16	3	5	8	6	5	1	
		Total no. of families	7	4	4	2	1	4	3	2	1	
	Non-volant	Total no. of individuals	148	64		7	33	21	25	19		1
		Total no. of species	18	13		4	10	9	8	7		1
		Total no. of families	5	5		3	4	3	5	3		1
Coastal forest	Volant	Total no. of individuals	211	33	26	1	7	6	1			
		Total no. of species	12	6	5	1	5	3	1			
		Total no. of families	5	5	3	1	2	2	1			
	Non-volant	Total no. of individuals	99	43			24	13	21	2		
		Total no. of species	8	6			6	4	5	1		
		Total no. of families	5	4			4	3	3	1		
Insular forest	Volant	Total no. of individuals	81	19	16				2			
		Total no. of species	9	7	7				2			
		Total no. of families	4	3	3				2			
	Non-volant	Total no. of individuals	80	27			2	15	13	11		3
		Total no. of species	10	7			2	5	4	2		1
		Total no. of families	4	4				3	3	1		1

## 2 Experimental Design, Materials and Methods

Field samplings on small mammals were conducted in three different habitats in Terengganu including the mixed dipterocarp forest, coastal forest and insular forest off coast islands from February 2015 until February 2016. The description of these habitats has been described by [2], [3]. A total of 10 standard mist-nets and two sets of four-bank harp trap, and 100 cages baited traps were used to capture bats and non-volant small mammals, respectively [1], [2], [3], [4], [5], [6], [7], [8]. The captured small mammals were identified, and standard morphological measurements and weights were recorded. Then the samples were sorted to live sample or voucher species to enable appropriate ectoparasite extracting method to be used.

A fine tooth-comb was used to remove all the ectoparasite that attached on the host animal coat. Ectoparasite dropped during combing were collected by using the wet sharpen applicator stick and preserved in the collecting vials containing 70 % ethanol separately for each host small mammals. The vials were labelled with sufficient information and brought back to the laboratory for species identification. The ectoparasites were prepared for mounting on slide and identified according to their taxonomic groups which are mesotigmatid mite [9,10], chiggers [11,12], ticks [13] and insect ectoparasite (fleas, lice and bat flies) [14,15]. The degree of specialisation among ectoparasite species was analysed using R version 3.3.2 (R core team 2016) and the package bipartite 2.08 [16] following [17].

## Appendix Supplementary materials

Image, application 1

Image, application 2
